# Anti-oxidant effect of heme oxygenase-1 on cigarette smoke-induced vascular injury

**DOI:** 10.3892/mmr.2015.3722

**Published:** 2015-05-04

**Authors:** GENHUAN YANG, YANCHUAN LI, WEI WU, BAO LIU, LENG NI, ZHANQI WANG, SHIYING MIAO, LINFANG WANG, CHANGWEI LIU

**Affiliations:** 1 Department of Vascular Surgery, Peking Union Medical College Hospital, Peking Union Medical College and Chinese Academy of Medical Sciences, Beijing 100730, P.R. China; 2 State Key Laboratory of Medical Molecular Biology, Institute of Basic Medical Sciences, Peking Union Medical College and Chinese Academy of Medical Sciences, Beijing 100005, P.R. China

**Keywords:** heme oxygenase-1, smoking, vascular, oxidative stress

## Abstract

Cigarette smoking, a major independent risk factor of atherosclerosis, can cause oxidative and inflammatory damage of vascular tissue. Heme oxygenase-1 (HO-1) is an endogenous cytoprotective enzyme with an anti-oxidant role in cells. The aim of the present study was to investigate whether HO-1 was able to protect vascular and endothelial cells from the oxidative damage induced by cigarette smoking. It was observed that cigarette smoking was able to induce the generation of the reactive oxygen species (ROS) in carotid arteries of rats. Hemin, a widely used HO-1 inducer, was able to reduce the generation of ROS. In addition, when human umbilical vein endothelial cells (HUVECs) were cultured in the serum of smoking rats, this was able to increase ROS, and the protective effect of hemin was also observed in this system. In conclusion, the present study demonstrated that cigarette smoking causes oxidative damage of vascular cells and HUVECs by inducing the generation of ROS, while HO-1 has an anti-oxidant effect in this course. This also implied that hemin, an inducer of HO-1, may have potential therapeutic applicability in the prevention of vascular diseases caused by cigarette smoking.

## Introduction

Cigarette smoking is the leading cause of preventable morbidity and mortality in the world (1). Cigarette smoke contains more than 5,000 chemicals (2,3), of which over 150 are hazardous smoke components contributing to the pathogenesis of a diversity of diseases (4). For example, cigarette smoke contains a large number of oxidizing chemicals, which can enter the blood and cause great damage to endothelial cells (5,6), which is a major independent risk factor of atherosclerotic vascular disease, including coronary artery disease, peripheral vascular disease and stroke. Smoking can weaken the anti-oxidant defense system and smokers have lower plasma levels of anti-oxidants (7,8). There is also evidence to support that administration of anti-oxidants such as vitamin C may improve endothelium-dependent responses in chronic smokers (9).

Heme oxygenase-1 (HO-1) is an endogenous cytoprotective enzyme that is induced ubiquitously in response to oxidative stress (10). The main function of HO-1 is to cleave heme to form carbon monoxide (CO), free iron and biliverdin (11). The cytoprotective effect of HO-1 may have several distinct underlying mechanisms, including the degradation of heme to the antioxidant bilirubin, the co-ordinate induction of ferritin, which chelates free iron, and release of CO, which exerts significant anti-inflammatory and anti-apoptotic effects (12,13). HO-1 can be induced by numerous oxidizing agents and stimuli, including ultraviolet radiation, heavy metals, cytokines and heme/hemoglobin (14,15). It is now well established that HO-1 can provide anti-oxidation and cytoprotection in *in vitro* and *in vivo* systems. HO-1-deficient mice were shown to develop severe iron deposition in the kidney and liver and exhibit tissue injury, chronic inflammation and oxidative damage of macromolecules (16). The first human case of HO-1 deficiency was also reported, characterized by iron deposition, growth retardation, anemia and vulnerability to oxidative stress (17). There is convincing evidence indicating that HO-1 can protect the vasculature against remodeling and atherogenesis (18,19). HO-1 is currently regarded as a novel therapeutic target in the treatment of vascular disease. There are several strategies that can be used to target HO-1 in the vasculature. One promising approach is the use of pharmacological inducers. Heme and its synthetic analogues as potent inducers of HO-1 have been proved to protect against the development of vascular disease in numerous studies (13,20,21).

The present study was designed to investigate the importance of heme oxygenase-1 in the pathophysiology of smoking-induced oxidative damage to endothelial cells *in vitro* and arteries *in vivo*. HO-1 levels and reactive oxygen species (ROS) in the arteries of cigarette-smoking rats and the effects of the HO-1 inducer heme and the HO-1 inhibitor zincprotoporphyrin IX (ZnPP) on these parameters were assessed. Furthermore, the effect of serum from smoking rats on oxidative damage via ROS in human umbilical vein endothelial cells (HUVECs) was assessed. The present study indicated that hemin, an inducer of HO-1, may have potential therapeutic applicability in prevention of vascular disease in smokers.

## Materials and methods

### Animal model

The animal care and experimental protocol of the present study complied with the Animal Management Rules of the Chinese Ministry of Health and were approved by the Animal Care Committee of Peking Union Medical College (Beijing, China). Twenty male Sprague-Dawley rats with a mean weight of 350 g were used. The rats were provided and raised by the Laboratory Animal Center of Peking Union Medical College Hospital (Beijing, China). Rats were randomized into four groups (n=5 per group): Group A, normal rats injected with saline; group B, rats exposed to smoke and injected with saline; group C, rats exposed to smoke and injected with hemin (Sigma-Aldrich, St. Louis, MO, USA); and Group D, rats exposed to smoke and injected with ZnPP (Sigma-Aldrich).

Group B, C and D were exposed to the smoke of ten commercial cigarettes (13 mg tar and 1.2 mg nicotine per cigarette) each day for seven days according to the modified protocols of Hautamaki *et al* (22), whereas group A was not exposed to the smoke. Groups C and D were injected intraperitoneally with hemin (50 mg/kg) (20), a widely used HO-1 inducer or ZnPP (15 mg/kg) (20), an established inhibitor of HO-1, 48 h prior to exposure to smoke. Hemin and ZnPP were then injected every 48 h from two days prior to the first smoke exposure. Groups A and B received injections of equivalent volumes of saline.

### Vessel isolation and serum collection

Seven days after smoke exposure, animals were anesthetized by injection of sodium pentobarbital (Beijing Sunbiotech Co., Ltd, Beijing, China). The carotid arteries were isolated and cleaned from the surrounding tissue, then kept in liquid nitrogen for the measurement of HO-1. In addition, the contralateral carotid arteries were colleted, washed with sterile phosphate buffered saline (GE Healthcare Life Sciences, Logan, UT, USA) and maintained in endothelial cell medium (ECM; ScienCell Research Laboratories, San Diego, CA, USA) at 37°C in a humidified atmosphere containing 5% CO_2_, and for the measurement of ROS. The blood of the rats was collected from the abdominal aorta and kept at 4°C overnight. Then the serum was centrifuged at 3600 × g for 15 min at 4°C, kept at 56°C for 30 min to inactivate all the complement and was then stored at −20°C.

### Endothelial cell culture

HUVECs (ScienCell Research Laboratories, San Diego, CA, USA) were maintained in ECM at 37°C in a humidified atmosphere containing 5% CO_2_, and cells were grown to 80% confluence. ZnPP (10 *µ*M) (23), PBS and hemin (50 *µ*M) (23) were added to the medium, respectively, and 18 h later, HO-1 levels in the three groups were detected using western blot analysis. The serum from group B (smoking rats) was added to the three groups of HUVECs, accounting for 10% of the total volume. After 3 h, the ROS of the three groups were quantified. As a control, HUVECs were incubated in the serum from group A (normal rats), and ROS were quantified.

### Western blot analysis

Carotid arteries kept in liquid nitrogen were ground with a pestle and mortar with 600 *µ*l cell lysis solution (62.5 mM Tris-HCl, 2% SDS and 10% glycerol; pH 6.8). Then the mixture was maintained at 100°C for 10 min and centrifuged at 10,800 × g for 15 min. The supernatant was collected and the concentration of the protein in the supernatant was measured using the bicinchoninic acid method (Thermo Scientific, Inc., Waltham, MA, USA). 20 *µ*g of total protein extraction sample was separated by 12% SDS-PAGE and transferred to a polyvinylidene difluoride membrane (EMD Millipore, Billerica, MA, USA). The membrane was blocked in 5% skimmed milk (Applygen Technologies, Inc., Beijing, China) in TBST (20 mM Tris-HCl, pH 7.6, 137 mM NaCl and 0.05% Tween-20) for 1 h at room temperature. The membranes were then washed three times with TBST and incubated with mouse primary monoclonal IgG antibodies against HO-1 (1:2,000; 610712; Becton Dickinson, Franklin Lakes, NJ, USA) overnight at 4°C. Following three washes, the membranes were incubated in goat anti-mouse immunoglobulin G-conjugated horseradish peroxidase (1:5,000; Beijing Zhongshan Jinqiao Biotechnology Co., Ltd, Beijing, China) for 1 h at room temperature. Then, the antigen-antibody complexes were detected by enhanced chemiluminescence (Beijing TransGen Biotech Co., Ltd, Beijing, China). Images were captured and the gray values of target proteins were analyzed by AlphaEase FC software, version 4.0 (Alpha Innotech Corporation, San Leandro, CA, USA).

### Measurement of reactive oxygen species (ROS)

The measurement of the intracellular ROS was based on the fact that ROS can convert non-fluorescent 2′,7′-dichlorofluorescein diacetate (2′,7′-DCFH-DA) into fluorescent dichlorofluorescein (DCFH). Cells or carotid arteries were incubated in serum-free medium with 2′,7′-DCFH-DA (1:3,000; Beyotime Institute of Biotechnology, Shanghai, China) at 37°C for 20 min. Samples were washed with serum-free medium three times, and cells were observed under the fluorescence microscope (DMI4000 B; Leica Microsystems, Wetzlar, Germany) and carotid arteries were observed under the IVIS Spectrum Imaging System (Caliper Life Sciences, Inc., Hopkinton, MA, USA). The IVIS Spectrum Imaging system is a high-sensitivity, *in vivo* imaging technology platform that enables fluorescence visualization and measurement of fluorescence intensity within a living organism in real time. In addition, the fluorescence intensity of cells and the bioluminescence (photons ×10^8^/sec/mm^2^/Sr) of carotid arteries were measured using a Synergy H1 Hybrid Multi-Mode Microplate Reader (BioTek Instruments, Inc., Winooski, VT, USA) and the IVIS Spectrum Imaging System, respectively. The fluorescence intensity represented the ROS content.

### Statistical analysis

Values are expressed as the mean ± standard error and analyzed using SPSS 17.0 software (SPSS, Inc., Chicago, IL, USA). Each experiment was performed at least three times. The Student’s t-test was used to analyze the data. P<0.05 was considered to indicate a statistically significant difference between values.

## Results

### Smoke exposure results in increased expression of HO-1 in carotid arteries or rats, and hemin can induce the expression of HO-1

**Figure 1:**
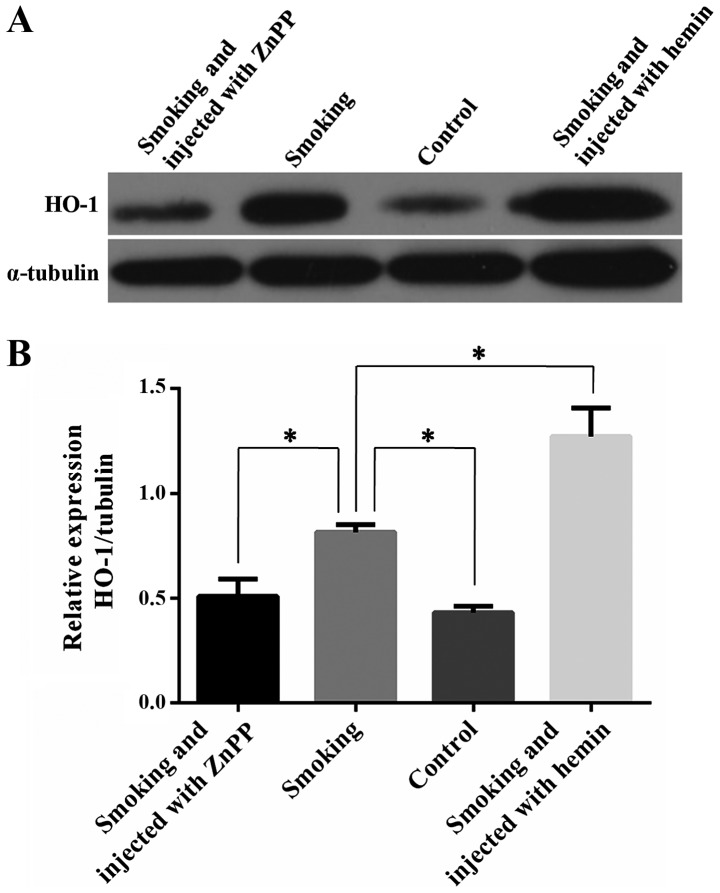
Expression of HO-1 in carotid arteries of rats from various treatment groups. (A) Western blot analysis showed that HO-1 levels in carotid arteries of smoking rats were higher than those of normal rats and smoking rats injected with hemin. Smoking rats injected with ZnPP exhibited the highest HO-1 levels. (B) Quantification of the western blot results. ^*^P<0.05; values are expressed as the mean ± standard deviation of 5 repetitions. ZnPP, zincprotoporphyrin IX; HO, heme oxygenase.

HO-1 expression in carotid arteries of rats in various treatment groups was detected by western blot analysis. Smoking induced the expression of HO-1 in carotid arteries, as the levels of HO-1 in the smoking group (group B) were significantly elevated compared with those in the control group (group A) (Fig. 1; P<0.05). As expected, the levels of HO-1 in smoking rats injected with HO-1 antagonist ZnPP (group D) were lower than those in the smoking group (P<0.05). The expression of HO-1 in smoking rats injected with hemin (group C) was obviously increased compared with that in the smoking group (P<0.05). These results suggested that smoking can cause the expression of HO-1 in carotid arteries and hemin can induce the expression of HO-1. In addition, ZnPP can successfully reduce the expression of HO-1 in carotid arteries.

### HO-1 protects from oxidative damage by decreasing smoke-induced ROS in vascular tissue

**Figure 2:**
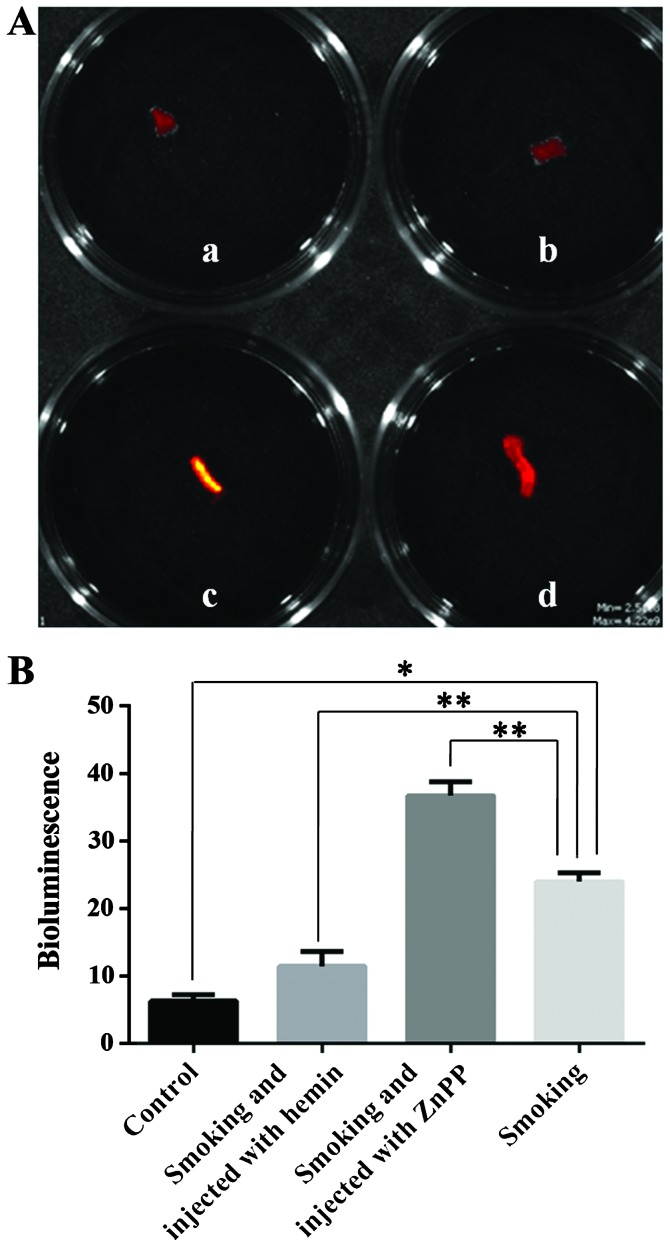
ROS in carotid arteries from rats in various treatment groups. (A) The IVIS Spectrum Imaging System was use to detect ROS in carotid arteries. The fluorescence intensity represents the content of ROS. (a) Control (normal rats); (b) smoking rats injected with hemin; (c) smoking rats injected with ZnPP; (d) smoking rats. (B) Quantification of the content of ROS through the IVIS Spectrum Imaging System. ^*^P<0.01, ^**^P<0.05, values are expressed as the mean ± standard deviation of 5 repetitions. ROS, reactive oxygen species; ZnPP, zincprotoporphyrin IX.

As is known, HO-1 can protect the vasculature against oxidative stress. The present study investigated whether smoke-induced oxidative stress may be attenuated in carotid arteries by increasing the expression of HO-1. An increase in endogenous ROS is one of the most commonly used indicators of oxidative stress. Therefore, the present study assessed the endogenous ROS levels to evaluate the state of oxidative stress. As shown in Fig. 2, the IVIS Spectrum Imaging System was employed to quantify the content of ROS in carotid arteries from the various groups by detecting the fluorescence intensity. The amount of ROS in the carotid arteries of the smoking group (group B) was higher than that in the control group (group A) (Fig. 2; P<0.01). Lower levels of ROS were detected in the carotid arteries of smoking rats injected with hemin (group C) compared with those in the smoking group (P<0.05). The carotid arteries of smoking rats injected with HO-1 antagonist ZnPP (group D) contained the highest amount of ROS (P<0.05). This indicated that the protective role of HO-1 in the oxidative damage induced by smoking was correlated with the generation of reactive oxygen species (ROS), suggesting a causative association. This further implied that increasing the expression of HO-1 may alleviate the oxidative damage of carotid arteries by reducing the amount of ROS.

### Incubation in serum from smoking rats increases the levels of endogenous ROS in HUVECs and HO-1 alleviates this effect

**Figure 3:**
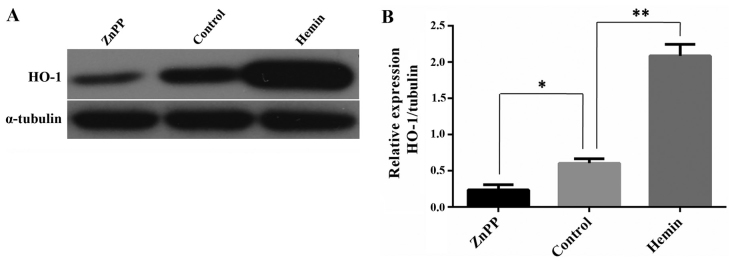
Expression of HO-1 in HUVECs pre-treated with ZnPP or hemin. (A) Western blot analysis showed that HUVECs pre-treated with hemin contained the highest levels of HO-1, while HUVECs pre-treated with ZnPP contained the lowest levels of HO-1. (B) Quantification of the western blot results. ^*^P<0.05, ^**^P<0.01, values are expressed as the mean ± standard deviation of 3 repetitions. ZnPP, zincprotoporphyrin IX; HO, heme oxygenase; HUVEC, human umbilical vascular endothelial cell.

**Figure 4:**
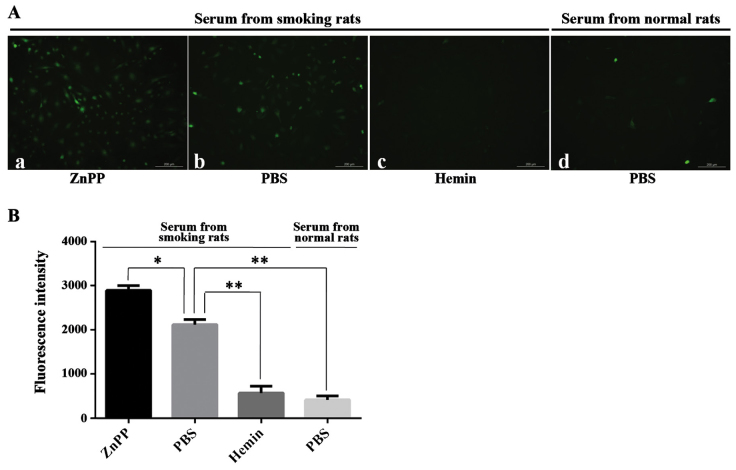
Content of ROS in HUVECs in various treatment groups. (A) HUVECs observed under the fluorescence microscope (scale bar, 200 *µ*m). The fluorescence intensity represents the content of ROS. (a) HUVECs pre-treated with ZnPP and incubated in the serum of smoking rats; (b) HUVECs pre-treated with PBS and incubated in the serum of smoking rats; (c) HUVECs pre-treated with hemin and incubated in the serum of smoking rats; (d) HUVECs pretreated with PBS and incubated in the serum of normal rats. (B) Quantification of the content of ROS through measurement of fluorescence intensity. ^*^P<0.05, ^**^P<0.01, values are expressed as the mean ± standard deviation of 3 repetitions. ZnPP, zincprotoporphyrin IX; PBS, phosphate-buffered saline; HUVEC, human umbilical vascular endothelial cell; ROS, reactive oxygen species.

As oxidative damage to vasculature, particularly to endothelial cells, contributes to the development of atherosclerotic vascular disease, the effects of sera from smoking rats on HUVECs, as well as the effect of HO-1 in this system, were assessed in the present study. First, expression of HO-1 in HUVECs pre-treated with hemin or ZnPP was assessed. The results showed that hemin obviously induced the expression of HO-1, while ZnPP depressed the expression of HO-1 compared with that in control cells treated with PBS (Fig. 3; P<0.05). Similar to the *in vivo* results, the cells pre-treated with PBS and incubated in the serum of smoking rats (group B) contained more ROS than the cells pre-treated with PBS and incubated in the serum of normal rats (group A) (Fig. 4; P<0.01). In addition, the ROS in cells pre-treated with hemin and incubated in the serum of smoking rats declined compared to those in cells pre-treated with PBS and incubated in the serum of smoking rats (P<0.01). The largest amount of ROS was detected in cells pre-treated with ZnPP and incubated in the serum from smoking rats (P<0.05). The results suggested that HO-1 may also function in the protection of HUVECs against the oxidative damage caused by serum from smoking rats by reducing the amount of ROS.

## Discussion

Smoking is one of the greatest contributors to vascular disease and a major cause of structural and functional changes of the vasculature (24). It is well known that oxidative stress and endothelial dysfunction are the main pathophysiological mechanisms linking cigarette smoking with vascular diseases (1,6,25). Thus, exploring ways to reduce smoke-induced oxidative stress in vasculature is an important area of research. Previous studies have demonstrated that HO-1 can protect against vascular constriction and proliferation (20–26). However, to the best of our knowledge, a direct protective effect of HO-1 on oxidative damage induced by cigarette smoking in vascular and endothelial cells has not been investigated to date.

The present study revealed that the expression of HO-1 in carotid arteries of smoking rats was markedly higher than that of normal rats. This result suggested that cigarette smoking may cause oxidative damage, which stimulates the tissue to produce HO-1 to resist the damage. Next, ROS, an indicator of oxidative stress, were assessed in carotid arteries from rats in the various treatment groups. The beneficial effects of HO-1 on smoke-induced oxidative stress were established by the use of either an HO-1 agonist or inhibitor. The results showed that the protective role of HO-1 in cigarette smoking correlated with the generation of ROS, suggesting a causative association. The finding that hemin can alleviate oxidative damage caused by cigarette smoking may have potential therapeutic applicability in the prevention of vascular diseases in smokers. Hemin, a HO-1 inducer, has been approved by the United States Food and Drug Administration for the treatment of acute porphyria and several other diseases (27). Importantly, a clinical study on a small subject cohort indicated hemin was able to stimulate the expression of human plasma HO-1 protein without adverse effects. However, further clinical studies must be employed on larger subject populations to establish optimal and safe administration schedules of hemin (28).

Cigarette smoking, as a risk factor for vascular diseases, mainly leads to oxidative stress and endothelial dysfunction (5,25). Therefore, preventing oxidative stress is of therapeutic interest. Endothelial injury is considered to be an important initiating event in the pathogenesis of atherosclerosis (28). Accumulating evidence supported the hypothesis that cigarette smoke can damage the endothelium (5,6). Therefore, the present study assessed the effect of serum from smoking rats on HUVECs pre-treated with hemin or ZnPP, as well as the effect of HO-1 in this system. It was directly demonstrated that cigarette smoking can cause oxidative damage of endothelial cells, which was caused by oxidative substances in the serum of smoking rats in the present experiment. The direct oxidative damage of endothelial cells by substances in the blood of smokers may be one of the underlying mechanisms of the vascular oxidative damage cause by cigarette smoking. In addition, the present study found that HUVECs pre-treated with hemin and incubated in the serum of smoking rats contained less ROS. This result suggested that HO-1 was able to protect endothelial cells from oxidative damage caused by cigarette smoking.

In conclusion, the present study found that i) the expression of HO-1 in carotid arteries of smoking rats was markedly higher than that of normal rats; ii) the serum of smoking rats can induce the oxidative stress in HUVECs; iii) HO-1 can protect vascular cells and HUVECs from smoke-induced oxidative stress by reducing endogenous ROS; and iv) hemin, a HO-1 inducer, can alleviate oxidative damage caused by cigarette smoking. Thus, the *in vivo* and *in vitro* findings of the present study showed a positive effect of HO-1 on oxidative damage caused by cigarette smoking and strongly supported the notion that hemin has a potential therapeutic applicability in the prevention of vascular diseases in smokers.
